# Seed type and origin‐dependent seedling emergence patterns in *Danthonia californica*, a species commonly used in grassland restoration

**DOI:** 10.1002/pei3.10105

**Published:** 2023-04-01

**Authors:** S. Holden Jones, Paul B. Reed, Bitty A. Roy, William F. Morris, Megan L. DeMarche

**Affiliations:** ^1^ School of Life Sciences University of Hawai'i at Mānoa Honolulu Hawaii 96822 USA; ^2^ Institute for Applied Ecology Corvallis Oregon 97333 USA; ^3^ Institute of Ecology and Evolution University of Oregon Eugene Oregon 97403 USA; ^4^ Biology Department Duke University Durham North Carolina 27708 USA; ^5^ Plant Biology Department University of Georgia Athens Georgia 30606 USA

**Keywords:** climate warming, common garden, local adaptation, nonlocal

## Abstract

*Danthonia californica* Bolander (Poaceae)is a native perennial bunchgrass commonly used in the restoration of prairie ecosystems in the western United States. Plants of this species simultaneously produce both chasmogamous (potentially outcrossed) and cleistogamous (obligately self‐fertilized) seeds. Restoration practitioners almost exclusively use chasmogamous seeds for outplanting, which are predicted to perform better in novel environments due to their greater genetic diversity. Meanwhile, cleistogamous seeds may exhibit greater local adaptation to the conditions in which the maternal plant exists. We performed a common garden experiment at two sites in the Willamette Valley, Oregon, to assess the influence of seed type and source population (eight populations from a latitudinal gradient) on seedling emergence and found no evidence of local adaptation for either seed type. Cleistogamous seeds outperformed chasmogamous seeds, regardless of whether seeds were sourced directly from the common gardens (local seeds) or other populations (nonlocal seeds). Furthermore, average seed weight had a strong positive effect on seedling emergence, despite the fact that chasmogamous seeds had significantly greater mass than cleistogamous seeds. At one common garden, we observed that seeds of both types sourced from north of our planting site performed significantly better than local or southern‐sourced seeds. We also found a significant seed type and distance‐dependent interaction, with cleistogamous seedling emergence peaking approximately 125 km from the garden. These results suggest that cleistogamous seeds should be considered for greater use in *D. californica* restoration.

## Graphical abstract

We did not find any evidence for local adaptation in a common garden study using both cleistogamous and chasmogamous seeds of *Danthonia californica*, a bunchgrass species commonly used in restoration outplanting. We found that cleistogamous seeds significantly outperformed chasmogamous seeds and that northern seeds outperformed sourthern seeds. Our results and methods suggest that cleistogamous seeds should be considered for more frequent use in *D. californica* restoration.

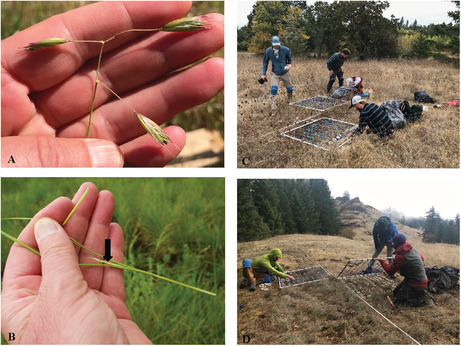

## 1 INTRODUCTION

Seed provenance is an important consideration for restoration practitioners seeking to re‐seed grassland ecosystems (Bischoff et al., 2006; Breed et al., 2018; Vander Mijnsbrugge et al., 2010). Seemingly minor differences in the fitness of seeds sourced from different populations can have profound effects on the establishment of focal plant populations at an ecosystem scale (Middleton et al., 2010; Seifert & Fischer, 2010). In 2020, the United Nations declared 2021–2030 the “Decade on Ecosystem Restoration” (UNEP and FAO, 2020). Grasslands have high potential for restoration under this declaration, but careful planning is needed to ensure long‐term success (Dudley et al., 2020). To achieve ambitious global restoration targets for grassland ecosystems, research on the relationship between seed provenance and plant fitness is urgently needed (Breed et al., 2018).

Restoration practitioners must consider the degree of local adaptation—the superior fitness of local genotypes—for plant species used in their projects. Populations under intense selective pressure are more likely to show local adaptation, providing them with a distinct “home” advantage over nonlocal populations at a given location (Breed et al., 2018; Joshi et al., 2001). Ecologists frequently use reciprocal transplant and common garden experiments to measure the degree to which local adaptation exists in plant populations (Hereford, 2010). Although intuitive from an evolutionary perspective, local adaptation is certainly not universal (Bischoff et al., 2006; DeMarche et al., 2019; Leimu & Fischer, 2008). Approximately 70% of reciprocal transplant studies show local adaptation (Hereford, 2009; Leimu & Fischer, 2008), which likely depends upon three variables (Hereford, 2009): the difference in selection pressure between local and nonlocal genotypes (Schluter & Grant, 1984), the amount of gene flow between populations (García‐Ramos & Kirkpatrick, 1997; Kawecki & Ebert, 2004; Lenormand, 2002), and the genetic structure of each population (Linhart & Grant, 1996).

Differences in selection pressure between populations is often closely related to the distance separating the populations (Hereford, 2009; Leimu & Fischer, 2008). Theory predicts that as distance increases between populations, so too should the magnitude of local adaptation (García‐Ramos & Kirkpatrick, 1997; Joshi et al., 2001). The underlying logic is simple; genotypes proven to perform well in a site should continue to do so in the future, while genotypes sourced from elsewhere may not, especially as the differentiation between sites increases. Of course, local adaptation is not universal, and local maladaptation could be common, especially at small scales (Hereford, 2009). At intermediate distances, nonlocal genotypes could have an advantage due to pathogen escape, although pathogens are infrequently accounted for in demography studies (Mackin et al., 2021; Nelson, 2018). The degree of local adaptation thus varies among populations and can be difficult to predict (Galliart et al., 2019; Leimu & Fischer, 2008). Understanding how seedlings perform near and far from their maternal plants can help elucidate the degree of local adaptation in plant populations.

Another consideration for choosing seed sources for restoration is how to best maintain genetic diversity (McKay et al., 2005). Although most plant species produce a single type of seed, many exhibit seed heteromorphism—the production of multiple seed types. Nearly 700 angiosperm species exhibit cleistogamy, a breeding system that includes permanently closed, obligately self‐pollinated flowers (Culley & Klooster, 2007). The majority of these species are classified as dimorphically cleistogamous, producing seeds from both cleistogamous and chasmogamous (more typical, externally pollinated) flowers (Baskin & Baskin, 2017; Culley & Klooster, 2007). As such, cleistogamous seeds are likely to have less genetic diversity than their potentially outcrossed chasmogamous counterparts and could be more prone to inbreeding depression (Culley & Klooster, 2007; Culley & Wolfe, 2001; Thammina et al., 2018). Cleistogamous seeds also typically disperse much shorter distances than chasmogamous seeds (Auld & Rubio de Casas, 2013; Baskin & Baskin, 2017; Culley & Klooster, 2007; Schoen & Lloyd, 1984), and average seed weight can differ substantially between the two types (Cheplick, 2023; Waller, 1982). There are, however, several evolutionary advantages to cleistogamy, including insurance in the absence of external pollination, the reduced energy cost of production, and the retention of locally adapted gene complexes (Baskin & Baskin, 2017; Culley & Klooster, 2007; Schoen & Lloyd, 1984). Indeed, a review of field and lab studies comparing the germination of cleistogamous and chasmogamous seeds found that a higher proportion of cleistogamous seeds germinated in two‐thirds of cases (Baskin & Baskin, 2017). The inherent differences in genetic diversity and dispersal between these two seed types suggest that chasmogamous seeds might be better suited for success in novel environments, while cleistogamous seeds may perform better in the immediate vicinity of their maternal plant (Culley & Klooster, 2007; Schoen & Lloyd, 1984).

To date, researchers have mostly recommended the use of locally sourced seeds for restoration (Bucharova et al., 2016; Vander Mijnsbrugge et al., 2010), despite approximately 30% of plant populations surveyed not showing local adaptation (Hereford, 2009). In these cases, stringent seed sourcing restrictions likely inhibit the genetic diversity of the restored population, which may have negative effects on the population's ability to respond to changing environmental conditions (Broadhurst et al., 2008; Miller et al., 2011). There is growing support for the use of nonlocal seeds sourced from populations that may be better adapted to future climatic conditions, as the use of climate‐adapted genotypes could facilitate the maintenance of ecosystem services and critical habitat structure (Broadhurst et al., 2008; Kreyling et al., 2011; Ramalho et al., 2017). Climate‐motivated translocation of seeds is controversial, however, as it relies on a series of assumptions that are difficult to test. These assumptions include that the seeds are sufficiently adapted to their local climate, that this climate adequately matches the future climate of the restoration site, that the nonlocal seeds will germinate and establish in a restored site under current conditions (Kreyling et al., 2011), and that climate is the most important driver of performance (DeMarche et al., 2019). While considering the long‐term effects of introducing novel genotypes, the emergence of nonlocal seedlings in a novel environment needs further study to ensure such an approach is feasible in the first place (Breed et al., 2018; Bucharova et al., 2016).

*Danthonia californica* Bolander (Poaceae), a perennial bunchgrass native to western North America, is commonly used in the restoration of prairie ecosystems in the Pacific Northwest, USA, where it is abundant (Buisson et al., 2006; Hayes & Holl, 2011; Pfeifer‐Meister et al., 2012; Stanley et al., 2011). Individuals produce both chasmogamous and cleistogamous seeds (Appendix A; A,B), the latter of which are enclosed within the stem. Cleistogamous seeds are difficult to remove manually, potentially contributing to their infrequent use in *D. californica* restoration (Hayes & Holl, 2011). Although mating system generally does not influence the degree of local adaptation across species (Hereford, 2010), many studies have shown different fitness and local adaptation patterns for conspecific seeds produced via different mating systems (Lovell et al., 2014; Rushworth et al., 2020; Schmitt & Gamble, 1990). However, discussion of the applied aspects of mating system‐dependent seed selection for restoration is relatively rare in the literature (Charlesworth, 2007; Coulter., 1914; Rushworth et al., 2020). *D. californica* thus provides an excellent opportunity to study the impacts of sourcing distance and mating system on local adaptation in an ecosystem restoration context.

**FIGURE 1:**
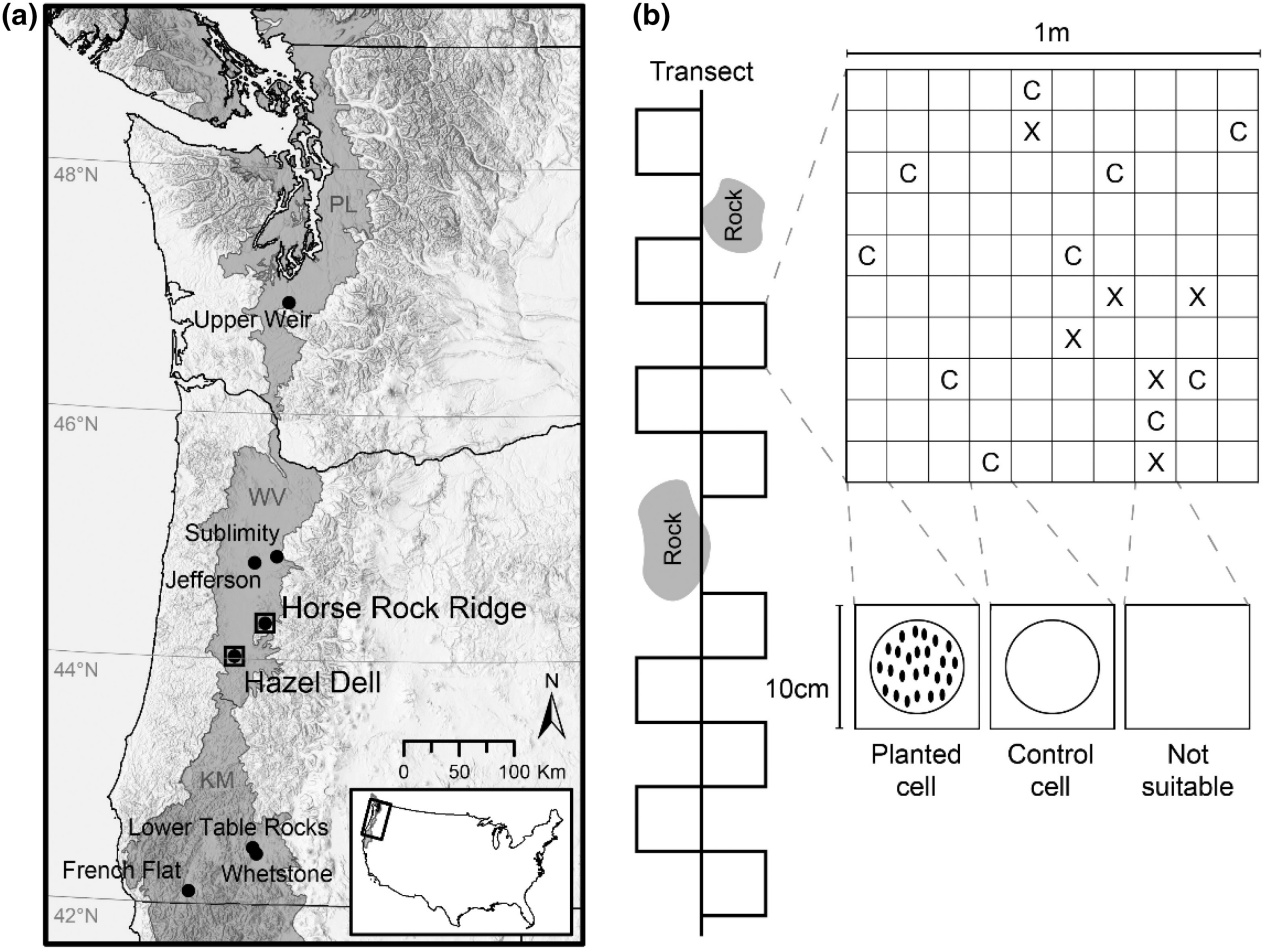
*Experimental design*. (a) Seed source locations (circles) and common garden (squares) of *D. californica* population locations within the Klamath Mountain (KM), Willamette Valley (WV) and Puget Lowland (PL) ecoregions. (b) Planting design showing alternating square meter grids along transects within natural *D. californica* populations at common garden sites. Each square meter grid was composed of 100 cells that were either planted, left as a control for background seedling emergence (c), or deemed not suitable for planting (X). See Appendix G: Table G4 for site location and environmental data and Appendix A for site photographs.

Here, we devised a common garden experiment using two gardens with very different environmental conditions but similar latitudes. We used both chasmogamous and cleistogamous seeds collected from eight natural populations of *D. californica* across a 534‐km latitudinal gradient in western Oregon and Washington, USA (Figure 1a). Our design allowed us to ask whether the effects of seed source origin (local vs. nonlocal) on seedling emergence are dependent on seed type and whether there are other factors about seed source origin, such as the distance or direction (north or south) from the common garden, latitude, or average seed weight, that help explain seedling emergence patterns across source populations.

We hypothesized the following: *H1a*: At each common garden, we predicted that both cleistogamous and chasmogamous seeds originating from that site (local seeds) would outperform seeds originating from other source populations (nonlocal seeds), regardless of whether the nonlocal seeds originated to the south or north of the common garden. *H1b*: However, we expected that the degree of local adaptation would depend on seed type. If inbreeding depression compromises local adaptation, then we would expect local chasmogamous seeds to outperform local cleistogamous seeds. Alternatively, if gene flow limits local adaptation, then we would expect local cleistogamous seeds to outperform local chasmogamous seeds. *H2*: Furthermore, we expected seedling emergence to decrease with increasing distance between source population and common garden, considering that the magnitude of local adaptation between common garden and source sites should increase as distance does. *H3*: Finally, we predicted that nonlocal seedling emergence would decrease with increasing latitude, as seeds sourced from southern populations would outperform seeds sourced from northern populations due to recent climate warming. Demographic studies of natural *D. californica* populations, including most of the populations studied here, revealed that population growth rate decreases with increasing latitude and that locally, the population growth rate decreases under warmer and drier conditions (DeMarche et al., 2021). Thus, it follows that the higher‐performing nonlocal seeds at the two common gardens should be those adapted to warmer and drier conditions (i.e., more southern populations).

## 2 MATERIALS AND METHODS

### 2.1 Experimental design

We selected two natural *D. californica* populations located outside of Eugene, Oregon, as common garden sites for our experiment. The first common garden, Hazel Dell (hereafter HD; N44.01979, W123.21823, 157 masl), is a seasonal wet prairie at the southern end of the Willamette Valley (Appendix A; C). The second common garden, Horse Rock Ridge (hereafter HR; N44.29877, W122.87984, 570 masl), is an exposed ridgeline in the Coburg Hills (Appendix A; D), which are foothills of the Cascade Mountains. While these two sites are close relative to the entire latitudinal range from which we sourced seeds (Figure 1a), they represent very different ecological extremes at approximately the same latitude: HD being a mesic, deep soil, low‐elevation site (Appendix A; C), and HR being a steep slope with dry, shallow soil, and at higher‐elevation (Appendix A; D). Outplanting at both common garden sites allowed us to assess seedling emergence across the habitat extremes of *D. californica* at a given latitude.

In June and July 2018, we collected approximately 50,000 cleistogamous and chasmogamous seeds (approximately 25,000 each) from natural *D. californica* populations at our two common garden sites and six other sites in the greater Klamath Mountain—Willamette Valley—Puget Lowland ecoregions (Figure 1a). Both seed types were collected from each maternal plant; both occur on the same reproductive stems. We collected 5–10 reproductive stems from each of 11–21 large maternal plants (median = 15) spaced at least 1 m apart and within a 250 m^2^ area at each population.

### 2.2 Seed preparation

**FIGURE B1:**
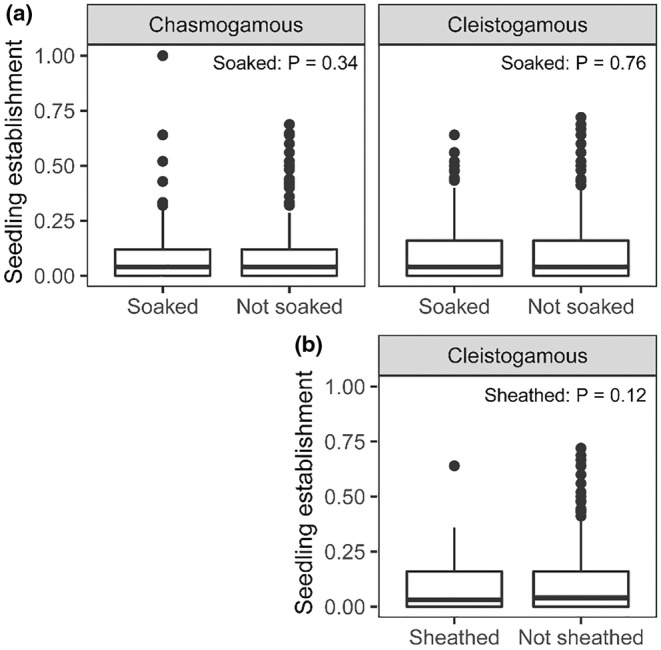
Neither soaked (a) nor sheathed (b) seed treatments affect the emergence of chasmogamous or cleistogamous seeds. The sheathed treatment is unique to cleistogamous seeds since sheathed refers to being enclosed within the stems.

Stems were stored in a cool, dry environment for no more than 90 days before we manually separated seeds by type. Chasmogamous seeds were shaken from inflorescences and manually extracted with forceps when necessary. We used three approaches to separate cleistogamous seeds from their stems, which did not impact cleistogamous seedling emergence (Appendix B: Figure B1). Once we extracted all seeds, we randomly weighed a subset of non‐soaked or sheathed chasmogamous and cleistogamous seeds (~20 each) from each maternal plant to calculate average seed weights by maternal plant.

### 2.3 Common garden experiment

We planted extracted seeds at HD on September 29th, 2018, and HR on September 30th, 2018. In areas with mature *D. californica* individuals already present, we aligned 15‐m transects with 1 m^2^ quadrats alternating every other side of the transect (Appendix A; C,D), avoiding areas where large rocks were present. Each 1 m^2^ quadrat was divided into 100 planting cells of 1 cm^2^ each (Figure 1b). If a cell was suitable for planting (i.e., with adequate soil and large adult plants absent), we installed 5.5 cm diameter, 1.25 cm deep PVC rings into the soil to define the planting area (Appendix A; C,D). We left approximately 10% of suitable planting cells undisturbed after ring installation as controls to allow for the detection of background *D. californica* seedling emergence from the natural population (Figure 1b). In each of the remaining planting cells, we planted 25 seeds of a given type and maternal plant in a predetermined randomized order, thereby randomizing planting location.

### 2.4 Seedling census

We censused seedling emergence at HD on April 12th, 2019, and at HR on April 17th, 2019. We counted *D. californica* seedlings in all rings, including unplanted control cells to measure background seedling emergence. Seedlings and rings were manually removed from sites in June 2019 to avoid genetic contamination of natural populations.

### 2.5 Statistical analyses

We used R version 4.0.2 for all analyses and visualization (R Core Team, 2021), using the packages ‘lme4’ (Bates et al., 2015) and ‘glmmTMB’ (Brooks et al., 2017) to fit mixed‐effect models, the package ‘DHARMa’ (Hartig, 2017) to examine residual plots and test for zero‐inflation and overdispersion, the ‘Anova’ function from the ‘car’ package (Fox & Weisberg, 2019) using type III sum of squares to test for significant model terms, the package ‘sjPlot’ (Lüdecke, 2021) to calculate marginal and conditional R^2^ values based on Nakagawa et al. (2017), and the package ‘emmeans’ (Lenth, 2020) to calculate model‐estimated marginal means and conduct Tukey's post‐hoc tests for significant differences between categorical variables with more than two levels. To accommodate the type III sum of squares, we set contrasts = c(“contr.sum”, “contr.poly”). We used the ‘ggplot2’ package for data visualization (Wickham, 2016).

We first tested background seedling emergence by modeling the number of seedlings in each cell, fitting the interaction of common garden (HD, HR) and cell type (planted, control) as fixed effects and quadrat as a random effect. To account for the fact that many cells contained zero seedlings, we tested for zero‐inflation and overdispersion and examined the normality of residuals in different versions of the model. We fit a standard Poisson model, a negative binomial model, and a Poisson model with the inclusion of an observation level random effect (Browne et al., 2005; Harrison, 2015), as well as a zero‐inflated Poisson model. Upon inspection of residual plots and homogeneity of variance, we ultimately chose the Poisson model with the observation level random effect as it best met model requirements and improved fit via a reduction in AIC.

To address our hypotheses, we fit a series of binomial logistic regressions that each included average maternal seed weight as a covariate as well as quadrat and maternal plant nested within source population as random effects. We used binomial models to account for the fact that the number of seeds planted was known for each ring. In the vast majority of planted rings, we sowed exactly 25 seeds. Of the 1608 total planted cells, 242 were planted with an amount other than 25 seeds (range: 6–32, median: 19). For hypotheses 1a and 1b regarding local adaptation, the model also included the three‐way interaction of garden, seed origin (local, nonlocal to the south of the garden, and nonlocal to the north of the garden), and seed type (chasmogamous; cleistogamous) as fixed effects. We then ran separate models for hypotheses 2 and 3 to explore the possible mechanisms that could explain the presence or absence of seed origin effects. These models addressed the distances between source populations and common gardens and the latitudes of the source populations. Both models included three‐way interactions of these terms with garden and seed type, and the distance model also included a quadratic distance term to allow for nonlinearity (to account for the possibility that neither the nearest nor farthest populations would have greatest seedling emergence). As mentioned above, we tested for zero‐inflation and overdispersion and examined the normality of residuals for all these models, fitting a standard binomial model, a zero‐inflated binomial model, and a standard binomial model with an observation level random effect. Again, we ultimately chose the models with the observation level random effect as this improved model fit in each case.

Finally, we tested whether average maternal seed weight, a covariate in all the seedling emergence models, could be explained by seed type, source population, or source population latitude. We fit two linear mixed‐effect models: the first including the interaction of source population and seed type as fixed effects and maternal plant as a random effect and the second taking the average weight across cleistogamous and chasmogamous seed types and fitting source population latitude as a fixed effect and source population as a random effect.

## 3 RESULTS

**FIGURE C1:**
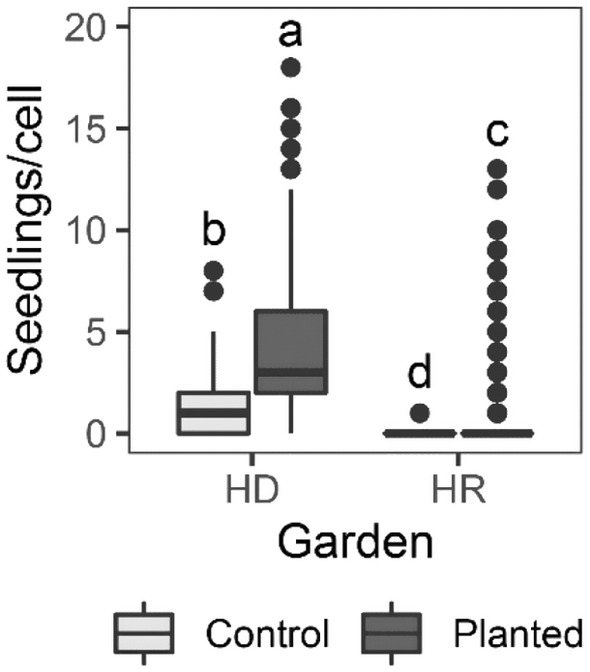
Background seedling counts at the two common garden sites (HD, Hazel Dell; HR, Horse Rock Ridge). Both control and planted cells had significantly more seedlings at HD than HR. Background counts at HD averaged 1.5 seedlings per cell and was negligible (~0.01 seedlings per cell) at HR. Planted cells averaged 3.9 and 0.6 seedlings per cell at HD and HR, respectively. Letters denote statistically significant differences between groups based on Tukey's post‐hoc tests (*p* < .05).

Seedling counts were greater at HD than at HR and were significantly greater in planted cells than in unplanted control cells at both sites (Appendix C: Figure C1; garden × cell type: *p*‐value = .018). While background (unplanted) seedlings were essentially negligible at HR (0.01 mean seedlings per unplanted control cell), there was a mean of 1.5 seedlings per control cell at HD.

**FIGURE 2:**
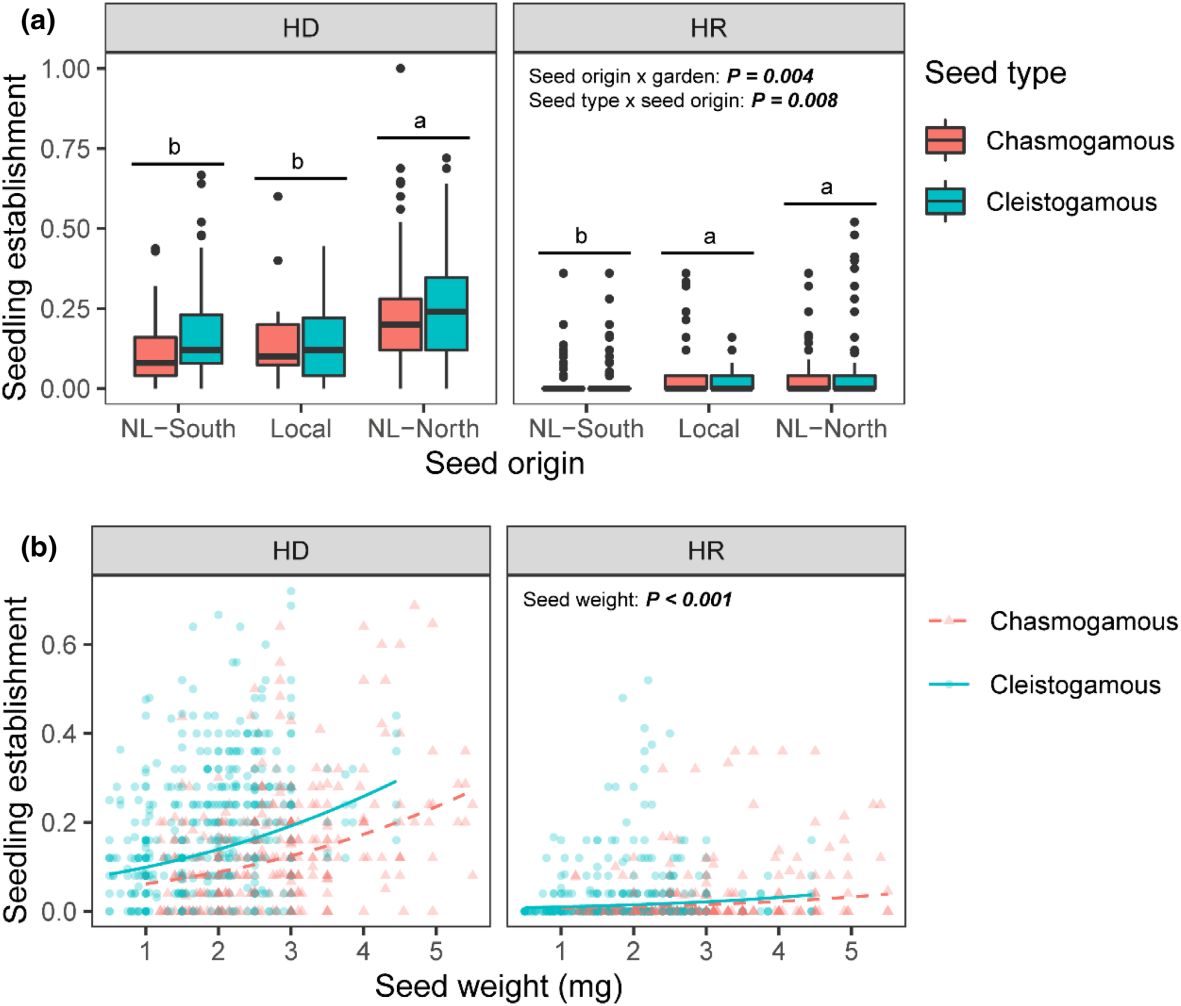
(a) Effect of seed origin on seedling emergence is dependent on garden: Nonlocal‐northern seeds (NL‐North) outperform both local and nonlocal‐southern seeds (NL‐South) at the Hazel Dell (HD) garden, while nonlocal‐northern and local seeds outperform nonlocal‐southern seeds at Horse Rock (HR) (letters denote differences based on Tukey's post‐hoc tests; *p* < .05). Across gardens, the effect of seed type on seedling emergence is dependent on seed origin: nonlocal cleistogamous seeds outperform nonlocal chasmogamous seeds (*p* < .05), whereas there are no significant differences between local cleistogamous and local chasmogamous seeds (*p* > .05). (b) Seed weight is also a significant positive predictor of emergence proportions across gardens. Predicted lines are averaged across seed origins. See Appendix G: Table G1 for complete model results.

**FIGURE D1:**
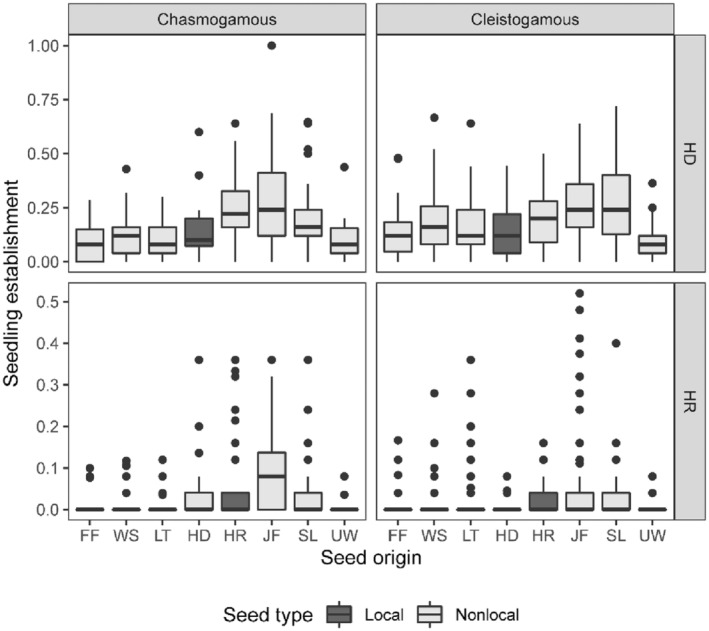
Seedling emergence at the two gardens displayed as a function of source population and seed type. Source populations are organized from left to right by increasing latitude. FF, French Flat; HD, Hazel Dell; HR, Horse Rock Ridge; JF, Jefferson; LT, Lower Table Rocks; SL, Sublimity; UW, Upper Weir; WS, Whetstone.

While the effect of seed origin on seedling emergence depended on garden (seed origin × garden: *p* = .004), there was a lack of evidence for local adaptation: at HD, nonlocal seeds sourced to the north significantly outperformed both local (HD) and nonlocal seeds sourced to the south, whereas at HR, both local (HR) and nonlocal seeds sourced to the north significantly outperformed nonlocal seeds sourced to the south (Figure 2a). Across the two gardens, the effect of seed type on seedling emergence depended on seed origin (seed type × seed origin: *p* = .008): nonlocal cleistogamous seeds outperformed nonlocal chasmogamous seeds (regardless of source direction north or south), whereas there were no significant differences between local cleistogamous and local chasmogamous seeds (Figure 2a). The effect of seed type did not depend on garden (seed type × garden: *p* = .362) nor was there a significant three‐way interaction (seed type × seed origin × garden: *p* = .793; see Appendix G: Table G1 for complete model results). Appendix D: Figure D1 displays seedling emergence data at each garden by specific source populations.

**FIGURE E1:**
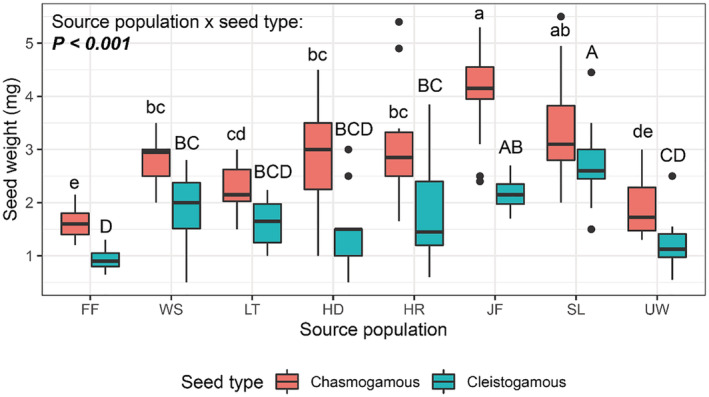
Maternal plant seed weight depends on seed type and source population. Cleistogamous seeds are universally lighter in weight than chasmogamous seeds across all source populations (*p* < .05), but the differences in source population depends on seed type (*p* < .001). Letters indicate significant differences among source populations separately for each seed type based on Tukey's post‐hoc tests. Source populations are organized from left to right by increasing latitude. FF, French Flat; HD, Hazel Dell; HR, Horse Rock Ridge; JF, Jefferson; LT, Lower Table Rocks; SL, Sublimity; UW, Upper Weir; WS, Whetstone.

**FIGURE F1:**
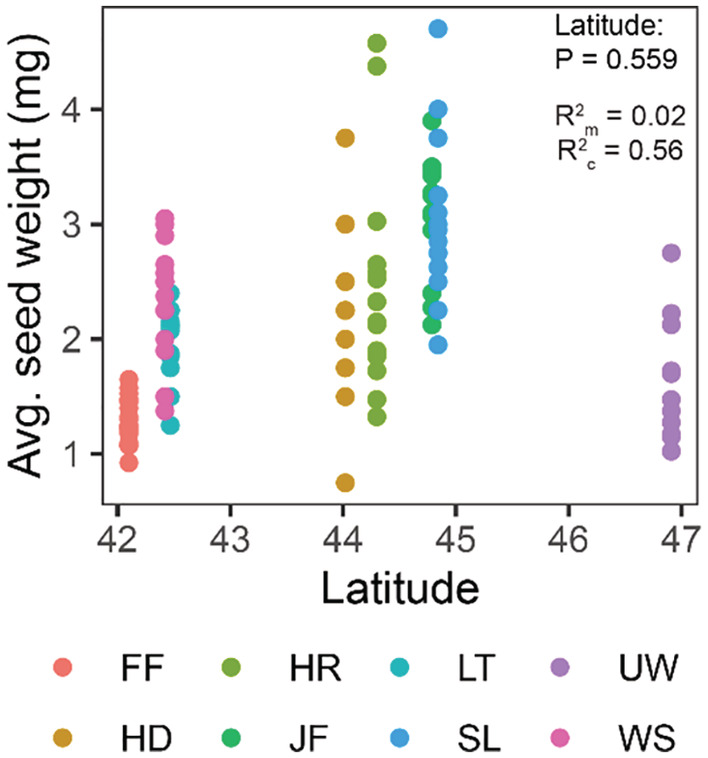
Average maternal plant seed weight (averaging cleistogamous and chasmogamous together) is not related to source population latitude. FF, French Flat; HD, Hazel Dell; HR, Horse Rock; JF, Jefferson; LT, Lower Table Rocks; SL, Sublimity; UW, Upper Weir; WS, Whetstone.

Across gardens, seed weight was a significant positive predictor of seedling emergence (*p* < .001; Figure 2b; Appendix G: Table G1). However, cleistogamous seeds showed greater seedling emergence despite being universally lighter weight than chasmogamous seeds across all source populations (*p* < .001; Appendix E: Figure E1). There was no significant relationship between average seed weight and source population latitude (*p* = .559; Appendix F: Figure F1).

**FIGURE 3:**
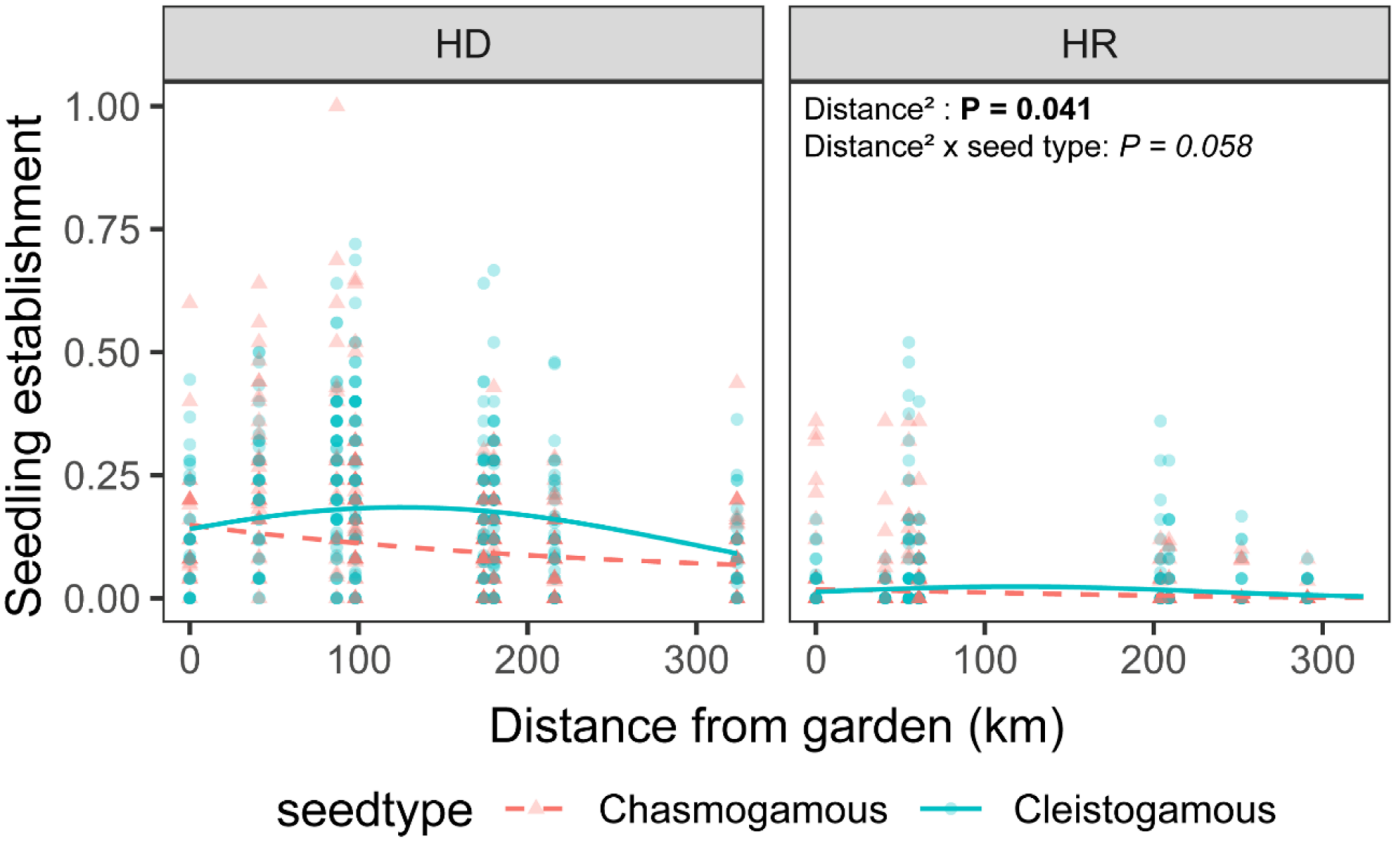
Across gardens, the distance effect is marginally dependent on seed type: chasmogamous seedling emergence decreases weakly as source distance from garden increases, whereas cleistogamous seeds show the highest probability of emergence when sourced between 100 and 200 km from the garden. See Appendix G: Table G2 for complete model results.

Across gardens, there was a significant nonlinear effect of distance between source population and garden on seedling emergence (distance^2^: *p* = .041), but this was marginally dependent on seed type (distance^2^ × seed type: *p* = .058): chasmogamous seedling emergence decreased weakly as distance from garden increased, whereas cleistogamous seeds showed the highest probability of emergence when sourced between 100 and 200 km from the garden (Figure 3). The effect of distance did not depend on garden (distance or distance^2^ × garden: *p* > .1) nor was there a significant three‐way interaction (distance or distance^2^ × seed type × garden: *p* > .5; Appendix G: Table G2). However, the distance effect was more noticeable at HD than at HR, likely due to the extremely low seedling emergence in general at HR.

**FIGURE 4:**
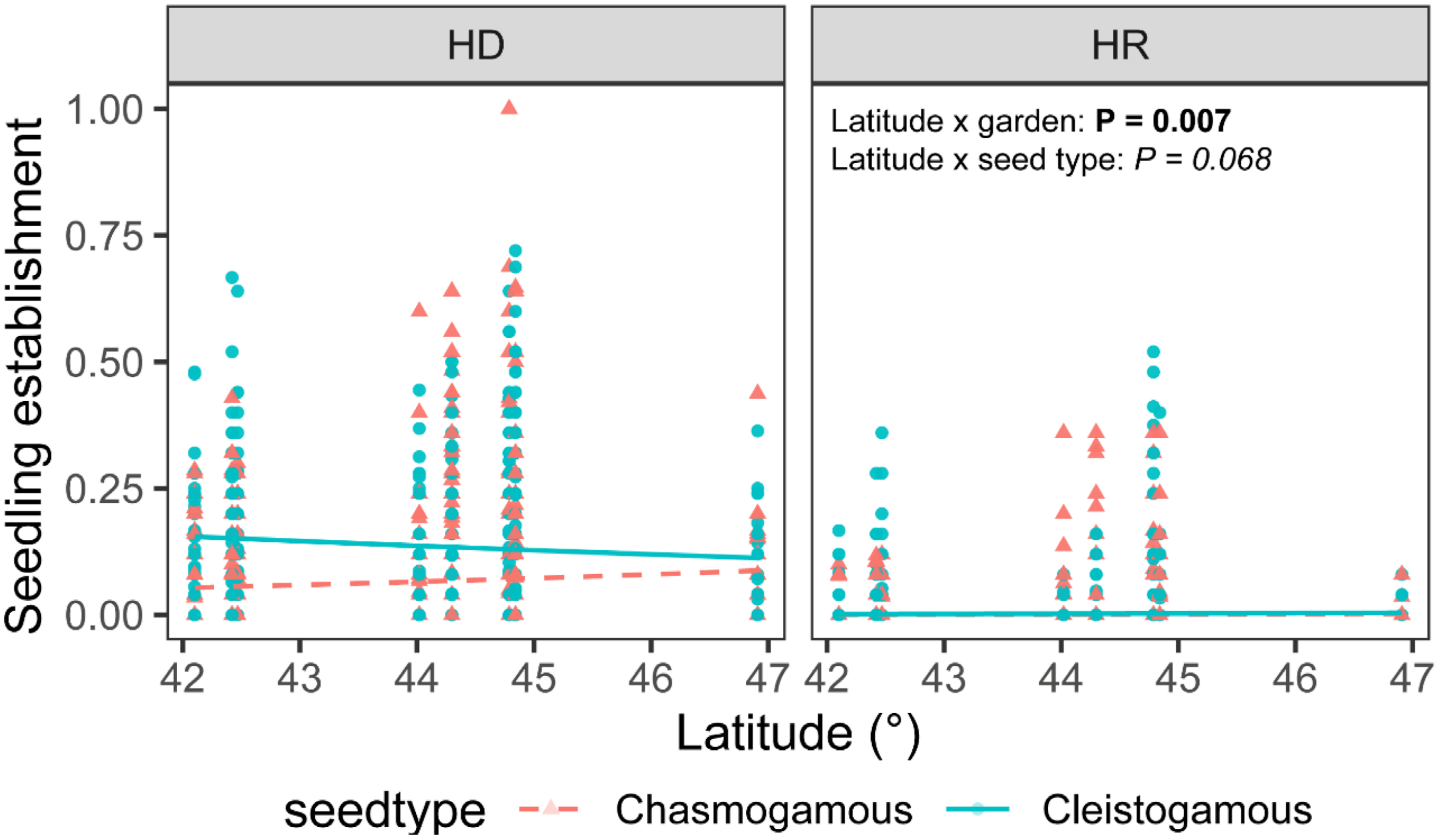
Source population latitude effects vary by garden and marginally by seed type. At Hazel Dell (HD), latitude exhibits a slight positive effect on emergence for chasmogamous seeds but a slight negative effect for cleistogamous seeds, whereas at Horse Rock (HR), the effects of latitude appear negligible. See Appendix G: Table G3 for complete model results.

The effect of source population latitude on seedling emergence depended on garden (*p* = .007) and marginally on seed type (*p* = .068), although the three‐way interaction was not significant (latitude × seed type × garden: *p* = .793; Appendix G: Table G3). At HD, latitude exhibited a slight positive effect on seedling emergence for chasmogamous seeds but a slight negative effect for cleistogamous seeds, whereas at HR, the effects of latitude appear negligible on both seed types (Figure 4).

## 4 DISCUSSION

Research on the degree of local adaptation in plant communities is urgently needed to inform ecosystem restoration efforts (Bischoff et al., 2006; Breed et al., 2018; Vander Mijnsbrugge et al., 2010). Here, we devised a common garden experiment using both cleistogamous and chasmogamous seeds of *D. californica* and expected to see a local advantage for seeds of both types. However, we did not find strong evidence for local seeds outperforming seeds sourced from other populations (nonlocal seeds) at either of our common garden sites. These results align with the findings of Hereford (2010) who, in a review of reciprocal transplant experiments, found that mating system does not generally influence local adaptation. Instead, we found that seeds of both types sourced from the north of our HD common garden performed significantly better than seeds sourced both locally and from more southern locations and that local seed performance did not differ from that of northern seeds in the HR garden, although both groups did outperform southern seeds.

An absence of local adaptation could stem from intermediate levels of either inbreeding or gene flow or a lack of strong divergent selective pressure on either of our common garden populations (Hereford, 2010; Lenormand, 2002). Another possibility for why we may not have seen local adaptation could be because our study looked exclusively at seedling emergence. By not measuring the viability of ungerminated seeds, and only measuring seedling emergence during one season, our study did not account for the role that seed dormancy may have had on the fitness of cleistogamous and chasmogamous seeds. Indeed, Carta et al. (2015) found that mating system and the degree of seed dormancy were closely related in *Hypericum elodes*. It is possible that cleistogamous and chasmogamous seeds of *D. californica* would show different dormancy patterns, which may provide additional fitness benefits to the maternal plant. Cheplick (2023) performed a reciprocal transplant experiment with *D. compressa*, a closely related eastern species, and found cleistogamous seeds to be five times more likely to produce seed‐bearing progeny than chasmogamous seeds over a 2‐year period. It is possible that our findings may have been different had we returned in a subsequent season, as ungerminated, viable seeds may still have established. Additionally, adaptation patterns likely come from the accumulation of small fitness advantages over a plant's life history, which are not always consistent with those of the establishment stage (Cheplick, 2023; Jin et al., 2020; Rice & Knapp, 2008).

### 4.1 Seed weight is an important predictor of seedling emergence

It is clear that seed weight is an important factor influencing the seedling emergence of both chasmogamous and cleistogamous seeds in our study. This is to be expected, as larger seed mass has long been linked to more energy investment and higher germination rates (Green & Hansen, 1969, Hendrix, 1984, but see Kitchen & Monsen, 1994), although the tradeoff between seed size and dispersal ability, seed number, and susceptibility to predation all mitigate the overall fitness benefits of increased seed size (Gómez, 2004; Gundel et al., 2012). Interestingly, Cheplick (2023) and others (Campbell et al., 1983; McNamara & Quinn, 1977) have found cleistogamous seeds to be heavier than chasmogamous seeds in grass species. Chasmogamous seeds of *D. californica* are heavier than cleistogamous seeds, but cleistogamous seeds generally outperformed chasmogamous seeds in our study.

Our hypothesis that nonlocal chasmogamous seeds would outperform nonlocal cleistogamous seeds was primarily motivated by the stark genetic differences between seed types (Culley & Wolfe, 2001; Kishikawa et al., 2019; Thammina et al., 2018), although expected differences in seed size, nutrient concentration, dispersibility, and germination requirements (reported for *D. spicata* in Clay & Antonovics, 1985) reinforce this prediction. We were surprised to find the reverse phenomenon to be true across all three source groups, with a significant seed type effect showing that non‐local, obligately selfed, smaller cleistogamous seeds significantly outperformed non‐local, potentially outcrossed, larger chasmogamous seeds. It is important to note however, that if the strong effects of seed mass were driven by environmental effects, genetically based differences in performance may have been obscured.

### 4.2 Seed‐type dependent distance effect

Even after controlling for seed mass, we saw a significant distance effect on cleistogamous seedling emergence. We found that cleistogamous seedling emergence peaked at approximately 125 km from the gardens, while chasmogamous seedling emergence declined slightly with increasing distance from the source population. This corroborates our prior finding that non‐local cleistogamous seeds germinate at a higher proportion than non‐local chasmogamous seeds at the HD garden, suggesting this pattern may be biologically significant. These results are surprising for the genetic and seed weight differences discussed previously, in addition to differences in the dispersal ability of the two seeds. In nature, cleistogamous seeds germinate almost exclusively within a single stem's length of the maternal plant (Holsinger, 2000), making them extremely unlikely colonizing mechanisms for *D. californica*. By contrast, chasmogamous seeds can disperse farther (Schmitt et al., 1985), making them better candidates to colonize novel environments (Culley & Klooster, 2007, but see Masuda & Yahara, 1992).

Contrary to prediction, the observed seed type and distance effect cannot be explained by genetic differences between cleistogamous and chasmogamous seeds. Indeed, our observation that cleistogamous seedling emergence success is improved, while chasmogamous seedling emergence success decreases away from the source population fundamentally dissatisfies the conditions for dimorphic cleistogamy maintenance at the population level as outlined by Culley and Klooster (2007). Mating system evolution theory predicts that cleistogamous and chasmogamous seeds with different dispersal abilities can be jointly maintained if their dispersal abilities differ, as they do for *D. californica* (Schoen & Lloyd, 1984). The near and far dispersal model for the selection of cleistogamy predicts that resource allocation will first be spent on the production of cleistogamous seeds—a so‐called *pessimistic* reproductive strategy—followed by chasmogamous seed production—an *optimistic* reproductive strategy (Schoen & Lloyd, 1984; Zeide, 1978). Our finding that cleistogamous seeds are generally more vigorous than their chasmogamous counterparts supports theory predicting that their investment should be prioritized and aligns with other studies that have found cleistogamous seeds to be generally superior (Baskin & Baskin, 2017; Cheplick & Quinn, 1982; Dyksterhuis, 1945; Schoen & Lloyd, 1984). It is possible that our experimental design, which planted 25 seeds from the same maternal plant within close proximity, may have unintentionally favored cleistogamous seedling emergence as these seeds are more likely to be subjected to sibling competition than farther dispersing chasmogamous seeds (Schoen & Lloyd, 1984). This may partially explain our finding that cleistogamous seeds were more vigorous than chasmogamous seeds. Although density‐dependent processes such as intraspecific competition could influence the expression of local adaptation, this is rarely addressed in local adaptation studies (Siepielski et al., 2016).

### 4.3 Latitude did not drive seedling emergence patterns

We expected to see higher seedling emergence of nonlocal seeds sourced from southern compared to northern sites due to recent warming patterns in the region (PRISM Climate Group, 2018) but were surprised to find the reverse pattern in our study. A comparison of locally sourced seeds to those sourced to the north and south of the gardens revealed that northern seeds performed significantly better than both local and southerly sourced seeds at HD, whereas both northern and local seeds outperformed southern seeds at HR, from which we observed almost no seedling emergence.

This pattern was not as easily detected in a latitude‐based seedling emergence model; as latitude increased, chasmogamous seedling emergence also increased slightly, while cleistogamous seedling emergence slightly decreased, which produced a significant latitude by seed‐type interaction. It is likely that the highly reduced seedling emergence probability of cleistogamous seeds at only our most northerly site created this negative latitudinal trend. This suggests there may be a northern limit for seed sourcing success at Willamette Valley planting locations.

Our prediction that southern seeds would outperform northern seeds also assumed that southern populations had adequately adapted to historically warmer conditions and that these conditions match recent climate warming. Given the rapid rate of climate change in the region and the high degree of habitat fragmentation throughout the Klamath Mountain and Willamette Valley ecoregions (Floberg et al., 2004), seeds sourced from the south may have been of a generally inferior quality than northern seeds, possibly linked to greater climate‐related environmental stresses at lower latitudes. Other studies have found that climate‐based environmental stresses lead to decreased germination (Oliveira et al., 2019; Ribeiro et al., 2021; Yi et al., 2019), which may help explain our finding that northern seeds outperform southern seeds at both common gardens.

It is unlikely that the opposing effects of latitude on chasmogamous and cleistogamous seeds observed in our continuous latitude model are biologically meaningful. Perhaps sourcing from more sites would allow us to examine the impact of latitude on seedling emergence more accurately. Indeed, the latitudinal range for *D. californica* extends from British Columbia to southern California, a latitudinal distance of approximately 2000 km (Darris & Gonzalves, 2008). The 534 km distance between our northern and southernmost sites is small relative to the entire species' latitudinal range but relevant to the range of latitudinal distances likely to be used during seed sourcing for restoration projects.

Because our study only lasted one growing season, we may have assessed seedling emergence during an abnormal winter that favored northern‐sourced seed, potentially making the detection of climate‐driven latitudinal fitness patterns unlikely (DeMarche et al., 2019; Galliart et al., 2019). Other local adaptation studies have found significant latitudinal responses (van Boheemen et al., 2019; Zhang et al., 2019), although they may not necessarily be tied to climate (DeMarche et al., 2021). Although a multi‐year analysis of seedling emergence was outside the scope of this study, multi‐year studies allow for a more thorough understanding of the mechanisms driving differential germination and establishment patterns (Cheplick, 2023; Merges et al., 2020; Pfeifer‐Meister et al., 2012; Rice & Knapp, 2008).

### 4.4 Implications for restoration

Restoration practitioners frequently use *D. californica* to reseed oak savanna and grassland ecosystems along the West Coast of the United States, although chasmogamous seeds have until now been used almost exclusively for this purpose (Hayes & Holl, 2011; Lindh, 2018; Maslovat, 2002). Our results suggest that cleistogamous seeds are generally more vigorous than chasmogamous seeds, and that northerly sourced seeds could establish at a higher proportion than southerly sourced seeds. We cannot, however, claim that higher seedling emergence probability will necessarily translate to greater fitness advantages over the plants' life history or that this pattern is likely to be replicated across the Willamette Valley. Despite finding a significant seed type effect at both planting locations, more common garden site replication would be needed to suggest a regional phenomenon (but see Bischoff et al., 2006, Miller et al., 2011, Gallagher & Wagenius, 2016). Future research at multiple planting sites and involving multiple life stages across several years is required to better address questions regarding seed translocation for restoration planting. Such a study should include multiple species of restoration importance in the Willamette Valley to investigate whether trends are consistent across species. This information would support ongoing efforts to create seed transfer zones of species used in the restoration of Willamette Valley ecosystems (Miller et al., 2011; Ramalho et al., 2017).

On a practical note, the methods we used to facilitate cleistogamous seed preparation substantially reduce the processing time for outplanting cleistogamous seeds (Appendix B: Figure B1). Restoration practitioners may benefit from incorporating cleistogamous seed planting as an insurance policy in the event of reduced chasmogamous seedling emergence in much the same way that the plants themselves do (Schoen & Lloyd, 1984; Zeide, 1978). This practice may be especially beneficial when local seed sourcing ability is limited.

Mackin et al. (2021) performed a detailed pathogen census using a subset of the seeds collected for this study and found that cleistogamous seeds have substantially lower pathogen loads than chasmogamous seeds. Although these differences seem like a possible explanation for our findings, we were unable to assess the impact pathogens had on in situ germination or seedling emergence. Thus, we cannot be certain that pathogen escape explains the intermediate distance advantage in cleistogamous seeds or the general overpeformance of cleistogamous seeds compared to chasmogamous seeds. Still, when seeding *D. californica* for restoration, practitioners should consider the pathogen communities of both source and planting sites, use cleistogamous seeds in addition to chasmogamous seeds, and consider sourcing cleistogamous seeds from more distant northerly populations than their chasmogamous counterparts.

## AUTHOR CONTRIBUTIONS

Conceptualization and methodology: all authors; formal analysis: Paul B. Reed, Megan L. DeMarche, William F. Morris, and S. Holden Jones; investigation: all authors; resources: Bitty A. Roy; data curation: S. Holden Jones; writing—original draft preparation: S. Holden Jones; writing—review and editing: all authors; seed collection: all authors; seed preparation: S. Holden Jones; visualization: Paul B. Reed; project administration: William F. Morris and Bitty A. Roy; funding acquisition: Bitty A. Roy and William F. Morris. All authors have read and agreed to the final version of the manuscript.

## CONFLICT OF INTEREST STATEMENT

The authors declare no competing interests.

## Data Availability

The data that support the findings of this study will be made openly available in Dryad Digital Repository upon acceptance.

## APPENDIX A

*Danthonia californica* experiment. (A) chasmogamous spikelets; (B) cleistogamous spikelets (at arrow); (C) garden at Hazel Dell; (D) garden at Horse Rock Ridge. Photos by B. A. Roy.

## APPENDIX B

### B.1 Methods

We experimented with seed processing techniques to facilitate cleistogamous seed preparation and decrease processing time for heteromorphic seed planting. This is necessary because cleistogamous seeds remain in the stem and are difficult to remove and separate. Most were manually extracted from stems by carefully slicing the stems so as not to damage seeds before removing them either individually or as a group when possible. However, we also soaked a random subset of stems containing both cleistogamous and chasmogamous seeds in room temperature tap water to facilitate cleistogamous seed extraction and sorting with a light table, hereafter referred to as soaked seeds. We compared these to an additional randomized subset of cleistogamous seeds that were still enclosed within the stem but separated into individual seed units, further facilitating seed processing, referred to as sheathed seeds.

We tested our seed processing techniques (soaked and sheathed) to see whether either technique affected emergence proportion (across the two gardens). We fit binomial logistic regressions separately for chasmogamous and cleistogamous seeds, with soaked as a fixed effect for chasmogamous and both soaked and sheathed as fixed effects for cleistogamous. Both models included random effects for quadrat, maternal plant nested within source population, and an observation level random effect for overdispersion.

### B.2 Results

Our sheathed and soaked manipulations did not affect emergence success for either cleistogamous or chasmogamous seeds (Appendix B: Figure B1), demonstrating that these methods can be used to make cleistogamous seed planting an accessible complement to chasmogamous planting. Although post‐soaked individual cleistogamous seed extraction is a laborious process when done by hand, planting groups of cleistogamous seeds within their intact stems is much less labor‐intensive. Our sheathed treatment demonstrates that planting cleistogamous seeds that are still enclosed within their stem can be an easy method to successfully establish cleistogamous *D. californica* seeds at a scale necessary for ecosystem restoration.

## APPENDIX G

**TABLE G1:** Binomial logistic mixed‐effect model results for the analysis of garden × seed origin × seed type on seedling emergence. Coefficient estimates are expressed as log‐odds values, using contrasts = c(“contr.sum”, “contr.poly”) to compare each group against the grand mean, where the intercept is the grand mean and others are deviations from that. For random effects: *σ* ^2^ = residual variance of logistic regression model; τ_00_ = between‐subject variance partitioned to each random effect in the model; ICC = intraclass correlation coefficient; *N* = number of levels to each random effect. OLRE = observation level random effect. Marginal *R* ^2^ provides the variance explained by the fixed effect(s) only, while Conditional *R* ^2^ provides the variance explained by both the fixed and random effects, as calculated in Nakagawa et al. (2017).

Predictors	Seed origin analysis		
	Odds ratios	CI	*p*
(Intercept)	0.02	0.01–0.02	<**.001**
garden1	3.26	2.67–3.98	<**.001**
seedorigin1	0.94	0.79–1.12	.497
seedorigin2	1.41	1.23–1.61	<**.001**
seedtype1	0.79	0.71–0.88	<**.001**
seedweight	1.48	1.33–1.64	<**.001**
garden1*seedorigin1	0.79	0.66–0.95	**.013**
garden1*seedorigin2	1.02	0.90–1.15	.779
garden1*seedtype1	0.98	0.89–1.07	.661
seedorigin1*seedtype1	1.26	1.07–1.48	**.005**
seedorigin2*seedtype1	0.95	0.85–1.06	.341
garden1*seedorigin1*seedtype1	0.88	0.75–1.04	.133
garden1*seedorigin2*seedtype1	1.07	0.95–1.19	.256
Random Effects			
*σ* ^2^	4		
τ_00OLRE_	0.71		
τ_00mother_	0.09		
τ_00quadrat_	0.18		
ICC	0.06		
*N*_quadrat_	23		
*N*_mother_	122		
*N*_OLRE_	1601		
Observations	1601		
Marginal *R* ^2^/Conditional *R* ^2^	0.318/0.361		

Bold denotes significant *p*‐values (< 0.05).

**TABLE G2:** Binomial logistic mixed‐effect model results for the analysis of garden × distance × seed type on seedling emergence proportions. Coefficients are expressed as log‐odds values, using contrasts = c(“contr.sum”, “contr.poly”) to compare each group against the grand mean, where the intercept is the grand mean and others are deviations from that. For random effects: *σ* ^2^ = residual variance of logistic regression model; τ_00_ = between‐subject variance partitioned to each random effect in the model; ICC = intraclass correlation coefficient; *N* = number of levels to each random effect. OLRE = observation level random effect. Marginal *R* ^2^ provides the variance explained by the fixed effect(s) only, while Conditional *R* ^2^ provides the variance explained by both the fixed and random effects, as calculated in Nakagawa et al. (2017).

Predictors	Geographic distance		
	Log‐odds	CI	*p*
(Intercept)	−3.72	−4.21 to −3.23	<**.001**
garden1	1.2	0.94 to 1.46	<**.001**
distance	0.24	−0.33 to 0.81	.41
distance^2^	−0.2	−0.40 to −0.01	**.041**
seedtype1	0.1	−0.09 to 0.30	.309
seedweight	0.33	0.22 to 0.44	<**.001**
garden1*distance	−0.15	−0.54 to 0.23	.426
garden1*distance^2^	0.11	−0.03 to 0.25	.113
garden1*seedtype1	−0.07	−0.24 to 0.11	.447
distance*seedtype1	−0.55	−0.91 to −0.19	**.003**
distance^2^*seedtype1	0.13	−0.00 to 0.26	.058
garden1*distance*seedtype1	0.11	−0.24 to 0.47	.533
garden1*distance^2^*seedtype1	−0.01	−0.14 to 0.12	.892
Random effects			
σ^2^	3.97		
τ_00OLRE_	0.68		
τ_00mother:source_pop_	0.07		
τ_00quadrat_	0.18		
τ_00source_pop_	0.04		
ICC	0.07		
*N*_quadrat_	23		
*N*_mother_	122		
*N*_source_pop_	8		
*N*_OLRE_	1601		
Marginal *R* ^2^/Conditional *R* ^2^	0.338/0.385		
AIC	5274.355		

Bold denotes significant *p*‐values (< 0.05).

**TABLE G3:** Binomial logistic mixed‐effect model results for the analysis of garden × latitude × seed type on seedling emergence proportions. Coefficient estimates are expressed as log‐odds values, using contrasts = c(“contr.sum”, “contr.poly”) to compare each group against the grand mean, where the intercept is the grand mean and others are deviations from that. For random effects: σ^2^ = residual variance of logistic regression model; τ_00_ = between‐subject variance partitioned to each random effect in the model; ICC = intraclass correlation coefficient; *N* = number of levels to each random effect. OLRE = observation level random effect. Marginal *R* ^2^ provides the variance explained by the fixed effect(s) only, while Conditional *R* ^2^ provides the variance explained by both the fixed and random effects, as calculated in Nakagawa et al. (2017).

Predictors	Latitude		
	Log‐odds	CI	*p*
(Intercept)	−5.88	−6.47 to −5.30	<**.001**
garden1	2.17	1.90 to 2.44	<**.001**
latitude	0.19	−0.07 to 0.45	.146
seedtype1	−0.48	−0.64 to −0.33	<**.001**
seedweight	0.64	0.44 to 0.84	<**.001**
garden1*latitude	−0.17	−0.29 to −0.05	**.007**
garden1*seedtype1	0.06	−0.06 to 0.17	.362
latitude*seedtype1	0.12	−0.01 to 0.24	.068
garden1*latitude*seedtype1	0.02	−0.11 to 0.14	.793
Random effects			
σ^2^	3.29		
τ_00OLRE_	3.74		
τ_00mother:source_pop_	0.22		
τ_00quadrat_	0.32		
τ_00source_pop_	0.1		
ICC	0.57		
*N*_quadrat_	23		
*N*_mother_	122		
N_source_pop_	8		
*N*_OLRE_	1601		
Marginal *R* ^2^/Conditional *R* ^2^	0.403/0.744		
AIC	26251.157		

Bold denotes significant *p*‐values (< 0.05).

**TABLE G4:** Site and transect location data for *D. californica* sources and common gardens. Common garden sites highlighted in bold.

SITE	Region	Transect	GPS start Lat	GPS start long	GPS end Lat	GPS end long	DistanceHR (km)	DistanceHD (km)	Latitude
Upper Weir	WAS	2	N 46.90931	W 122.70974	N 46.90932	W 122.70932	290.57	323.72	46.90908
Sublimity	COR	1	N 44.841218	W 122.767179	N 44.841263	W 122.76488	60.97	98.1	44.841218
Jefferson	COR	1	N 44.787292	W 123.018684	N 44.787167	W 123.017999	55.42	86.8	44.787292
**Horse Rock Ridge**	**COR**	**2**	**N 44.29877**	**W 122.87984**	**N 44.2988**	**W 122.87996**	**0**	**41.12**	**44.29804**
**Hazel Dell**	**COR**	**1**	**N 44.01979**	**W 123.21823**	**N 44.01955**	**W 123.21808**	**41.12**	**0**	**44.01979**
Lower Table Rocks	SOR	1	N 42.46811	W 122.94635	N 42.46787	W 122.94623	203.62	173.93	42.4681
Whetstone	SOR	2	N 42.41961	W 122.90685	N 42.41938	W 122.90685	208.95	179.7	42.4204
French Flat	SOR	1	N 42.10083	W 123.63493	N 42.10090	W 123.63479	251.93	216.04	42.1008
